# Global Public Interest and Seasonal Variations in Alzheimer's Disease: Evidence From Google Trends

**DOI:** 10.3389/fmed.2021.778930

**Published:** 2021-12-10

**Authors:** Yan-Mei Mao, Peng Wang, Xiao-Yu Wang, Dong-Qing Ye

**Affiliations:** ^1^Department of Epidemiology and Biostatistics, School of Public Health, Anhui Medical University, Hefei, China; ^2^Inflammation and Immune Mediated Diseases Laboratory of Anhui Province, Hefei, China

**Keywords:** Alzheimer's disease, geriatric medicine, global public interest, seasonal variations, Google Trends, neurodegenerative diseases

## Abstract

**Background:** As the world's population ages, Alzheimer's disease (AD), a common neurodegenerative disease, is a major challenge to human health in the future. Understanding the information needs on AD of the global public can contribute to the prevention and control of AD. The purpose of this study was to explore global public interest and seasonal variations in AD using Google Trends (GT).

**Methods:** GT was used to obtain relative search volume (RSV) of the keyword “Alzheimer's disease” in six English-speaking countries (Australia, New Zealand, the USA, the UK, Canada, and Ireland) and the world from January 2004 to December 2020. Cosinor analysis was applied to detect the seasonality of AD-related RSV. Time series plot was used to observe the trend of annual mean AD-related RSV. Globally, hot topics and top rising topics related to AD were also analyzed. In addition, we also explored the geographical distribution characteristics of AD-related RSV.

**Results:** AD-related RSV declined steadily from January 2004 to December 2013 and rose steadily from January 2014 to December 2020. Search popularity of AD is low in the southern hemisphere, compared to the northern hemisphere. Cosinor analysis showed that there were significant seasonal variations in AD-related RSV in six English-speaking countries (all *P* < 0.05). Interestingly, regardless of the hemisphere, peaks were observed in the winter months and trough in the summer months. Topics related to the characteristics and etiology of AD, early onset AD, AD-related associations, care of AD patients, and diseases that can easily be confused with AD had received special attention.

**Conclusions:** There is increasing global public interest for AD and a significant seasonal variation in AD. A better understanding of the seasonal variations and public interest of AD by governments, health workers and patients can contribute to the prevention, management, and treatment of AD.

## Introduction

Alzheimer's disease (AD), a progressive neurodegenerative disease characterized by damage or destruction of nerve cells in the parts of the brain involved in thinking, learning and memory, is now one of the top 10 causes of death globally, and new and existing cases are likely to increase as the population ages and the COVID-19 pandemic continues (1, 2). About 35.6 million people worldwide were living with dementia in 2010, and the number is expected to double every 20 years. In other words, in the absence of effective prevention and treatment measures, AD patients will reach 65.7 million by 2030 and 115.4 million by 2050 (3). In the United States, deaths from stroke, heart disease and HIV decreased between 2000 and 2019, while the number of deaths from AD reportedly increased by more than 145 per cent. According to the latest calculations, by 2021, there will be an estimated 6.2 million Alzheimer's patients aged 65 and older in the United States alone (1). Genome Wide Association Studies have revealed multiple pathways contributing to AD (1, 4). Genetic and environmental risk factors can work with the natural history of the disease to accelerate cognitive decline. AD is more common in older people over 65 years old, and the risk increases with age. Currently, none of the drugs used to treat Alzheimer's disease can effectively slow down or prevent the damage and destruction of neurons, and AD causes a heavy social burden (1).

In recent years, the Internet has become a popular medium for people seeking health-related knowledge and information for self-diagnosis (5). Based on the feedback analysis of Internet user searches, we can effectively predict the evolution of health-related events and human behavior. Google Trends (GT) is a publicly accessible tool for analyzing Web queries that has been used for analysis and prediction in a variety of diseases, including chronic obstructive pulmonary disease (COPD), Dengue, Acne, Melanoma (6–10), etc. A systematic review of 109 GT-related papers published between 2006 and 2019 found that GT analysis was mainly used for visualization, seasonality, correlation, prediction and modeling of diseases, with corresponding statistical methods for each category (8). A study in Taiwan showed that GT data temporally coincided with the number of new dementia cases and outpatient visits, and that GT could predict the incidence of dementia and dementia-related outpatient visits (11).

Physicians and investigators need to be aware not only of what is hot in AD science, but also of issues of public interest. This helps health policy makers and health professionals provide AD relevant information, such as health resources, nursing skills, and social welfare information, to millions of AD caregivers. Seasonal rhythms regulate a variety of physiological activities in the body, including brain functions such as mood and cognition (12, 13). It has been confirmed that the pathology of AD disrupts these rhythms (14), but so far there is little research data on the relationship between AD and seasonality and its biological relevance. To explore the relationship between AD and seasonality and its underlying biological mechanism can further deepen the understanding of the pathogenesis of AD. The purpose of this study was to understand the global interest and seasonal variations of AD based on GT, in order to provide clues for the prevention, management, and treatment of AD.

## Materials and Methods

GT is a data platform created by Google based on user search behavior. By analyzing billions of Google search results, it tells users how popular a particular keyword is in cyberspace at any given time, thus providing in-depth analysis of the topics users care about. GT analysis can be used as an effective complement to field epidemiological investigations and clinical trial studies. It's anonymized (no one is personally identified), categorized (determining the topic for a search query), and aggregated (grouped together). GT normalizes search data to make comparisons between terms easier. Each data point is divided by the total searches of the geography and time range it represents to compare relative popularity. The resulting numbers are then scaled on a range of 0–100 based on a topic's proportion to all searches on all topics. The relative search volume (RSV) value represents the search heat relative to the highest point in a given area and time, and does not represent the total amount of search. Details about GT can be found at the following website (15).

### GT Search and Data Collection

On June 2, 2021, AD-related RSV within Australia, New Zealand, the USA, the UK, Canada, and Ireland were downloaded between January 01, 2004 and December 31, 2020 under the Health category. These six countries are typical countries in the Southern and Northern Hemispheres and can reflect AD's Internet search patterns in the Southern and Northern Hemispheres. The six countries are English-speaking countries with high RSVs, with Australia and New Zealand in the southern hemisphere and the remaining four in the northern hemisphere. Furthermore, data on the global AD-related RSVs were also searched and downloaded. The details for our search were as follows: Time Range (2004/01/01–2019/12/31), Category (Health), and Search Type (Web Search). We used search topics rather than search terms to avoid different uses of uniform search terms that affected GT search popularity score. Moreover, from 2004 to 2020, AD related topics were extracted from GT for public interest analysis on a global scale.

### Public Involvement

The study was anonymous and the public was not directly involved in its design.

### Statistical Analysis

Cosinor analysis was conducted to detect whether there was any seasonality in AD, and *P* < 0.05 was considered statistically significant (9). Time series plot was used to observe the trend of annual mean AD-related RSV. Cosinor analyses and time series plots were conducted with the use of the “season” package in R version 3.5.2.

The cosinor analysis is hinged on a sinusoid $St=Acos\left(\frac{2\pi t}{c}-P\right)$, *t*=*1,…, n*. Where, *A* denotes amplitude of the sinusoid, *t* denotes time of each data point, *c* denotes length of the seasonal cycle (established at 12 for monthly data), *P* denotes phase of the sinusoid, which explains where the seasonal peak occurs, *n* denotes the total number of data points and *n* = 204 (12 months per year × 17 years) in this study. In cosinor analyses, both sine and cosine *p*-values were tested for statistical significance. If the recommended *p* < 0.05, the result would be considered as “true,” indicating the existence of seasonality. The Poisson model was applied, and the offset was used in cosinor functions in order to adjust the unequal number of days in the months.

## Results

### Annual Trends in AD-Related RSV

**Figure 1:**
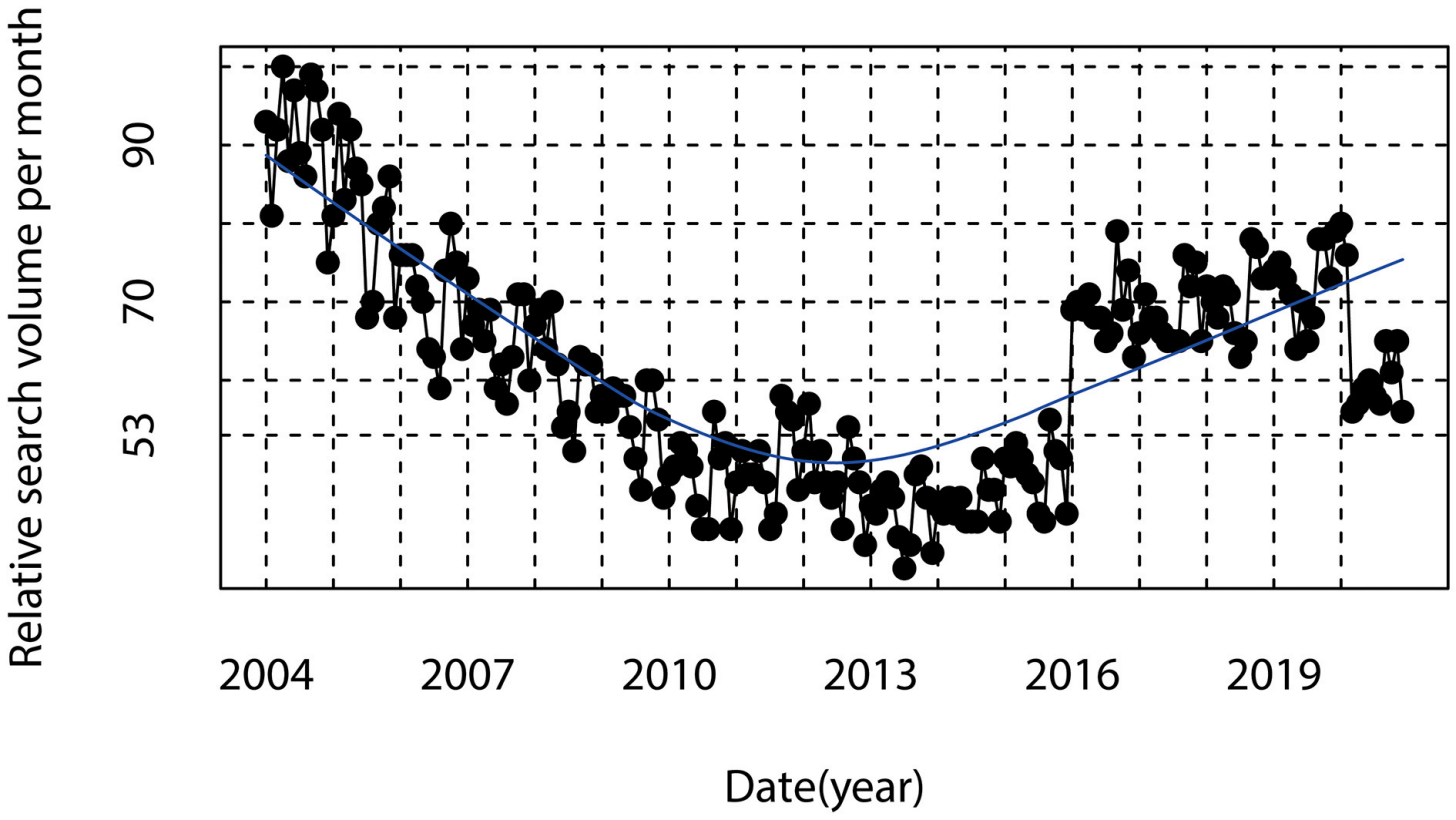
Time series plots for the Alzheimer's disease-related relative search volume worldwide.

The annual trends for AD-related RSV in GT are shown in Figure 1. From the trend line in Figure 1, we can find that AD-related RSV declined steadily from January 2004 to December 2013 and rose steadily from January 2014 to December 2020. Above results indicated the increasing interest of the global public in Internet search for AD in recent years.

### Interest by Region for the GT Topic “Alzheimer's Disease” From 2004 to Present

**Figure 2:**
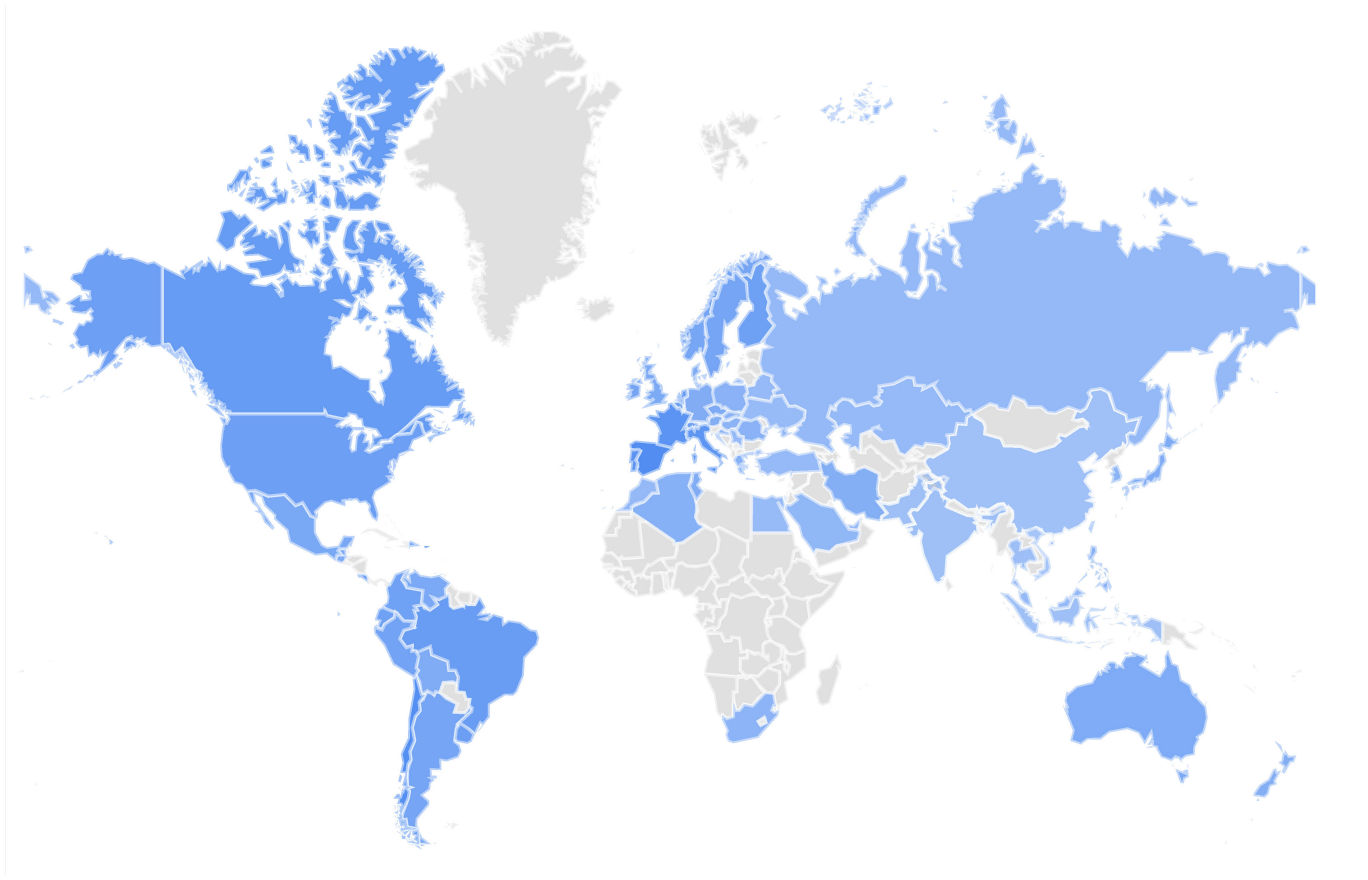
Interest by region for the Google Trends topic “Alzheimer's disease” from 2004 to present. The figure highlights that countries in the southern hemisphere are highly under-represented (color intensity outlines search interest). Data source: Google Trends (https://www.google.com/trends).

GT provides a number of features that may help identify geographic areas with low disease awareness and contribute to a better understanding of the current needs of AD patients and their caregivers. The color depth of the area in the diagram represents the Internet search popularity of the AD within that area. We can see that the search popularity of AD is low in the southern hemisphere (Figure 2).

### Related Topics of AD in GT

**Figure 3:**
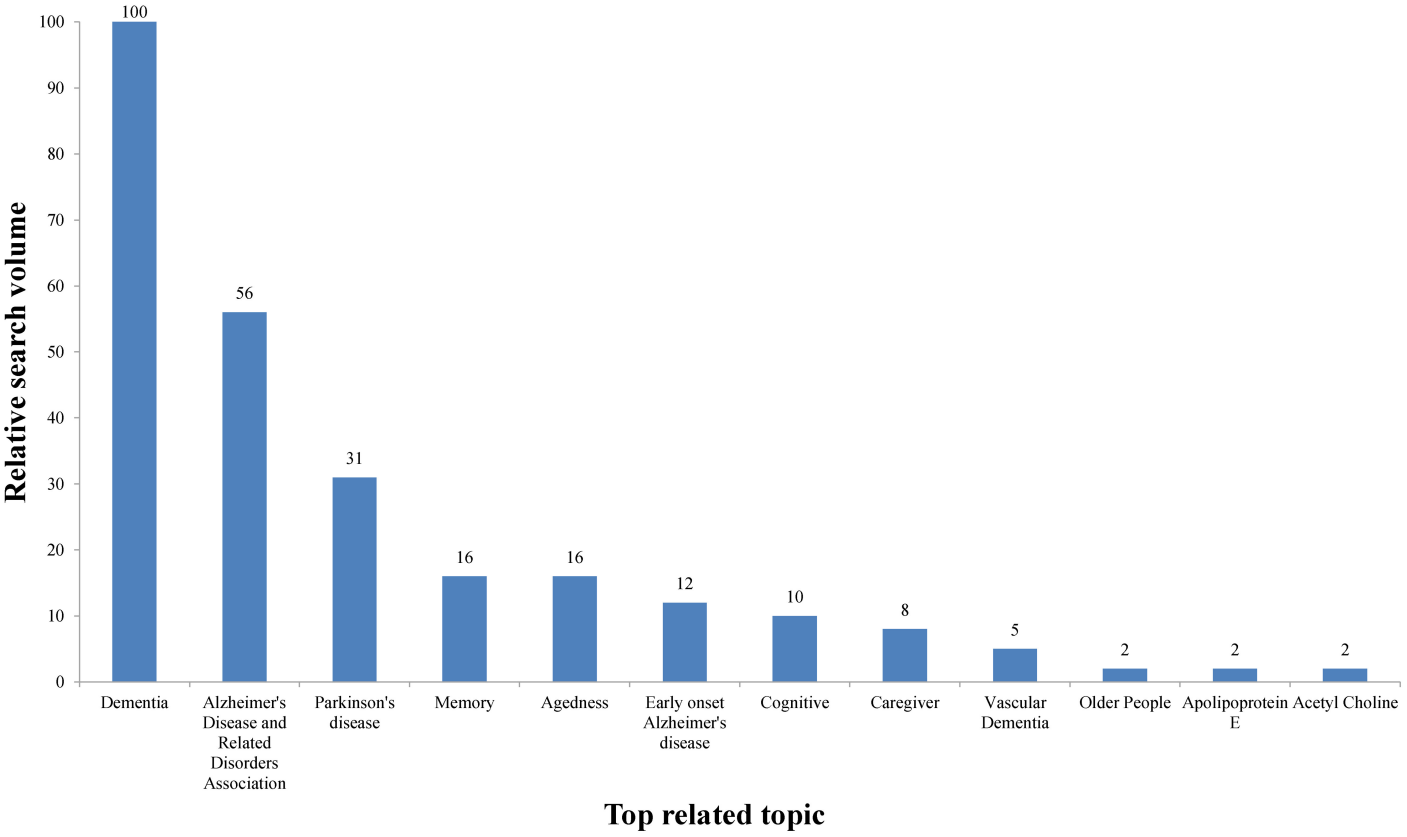
The histogram of the top related topics.

The most popular related topics were “Dementia” (RSV = 100), “Alzheimer's Disease and Related Disorders Association” (RSV = 56), “Parkinson's disease” (RSV = 31), “Memory” (RSV = 16), “Agedness” (RSV = 16), “Early onset Alzheimer's disease” (RSV = 12), “Cognitive” (RSV = 10), and others, in that order (Figure 3). The related topics with the largest increase in search frequency since the previous period are presented in Table 1. Regarding the rising related topics, “Vascular Dementia,” “Older People,” “Apolipoprotein E,” and “Acetyl Choline,” exhibited an increase over 5,000%, followed by “Alzheimer's Disease and Related Disorders Association”(*n* = 350), “Early onset Alzheimer's disease”(*n* = 300), “Cognitive” (*n* = 200), “Dementia” (*n* = 160), “Parkinson's disease” (*n* = 110), “Caregiver”(*n* = 100), and “Agedness”(*n* = 80).

**Table 1:** Relatively fast-growing topics regarding the term of “Alzheimer's disease.”

**Rank**	**Rising search topic**	**% Growth^a^**
1	Vascular dementia	Breakout^b^
2	Older people	Breakout^b^
3	Apolipoprotein E	Breakout^b^
4	Acetyl choline	Breakout^b^
5	Alzheimer's disease and related disorders association	350
6	Early onset Alzheimer's disease	300
7	Cognitive	200
8	Dementia	160
9	Parkinson's disease	110
10	Caregiver	100
11	Agedness	80

a *Term's growth is compared to the previous time period*;

b *Breakout is used for term search that grew by more than 5,000% compared to the previous period*.

### Seasonal Variations in AD-Related RSV

**Figure 4:**
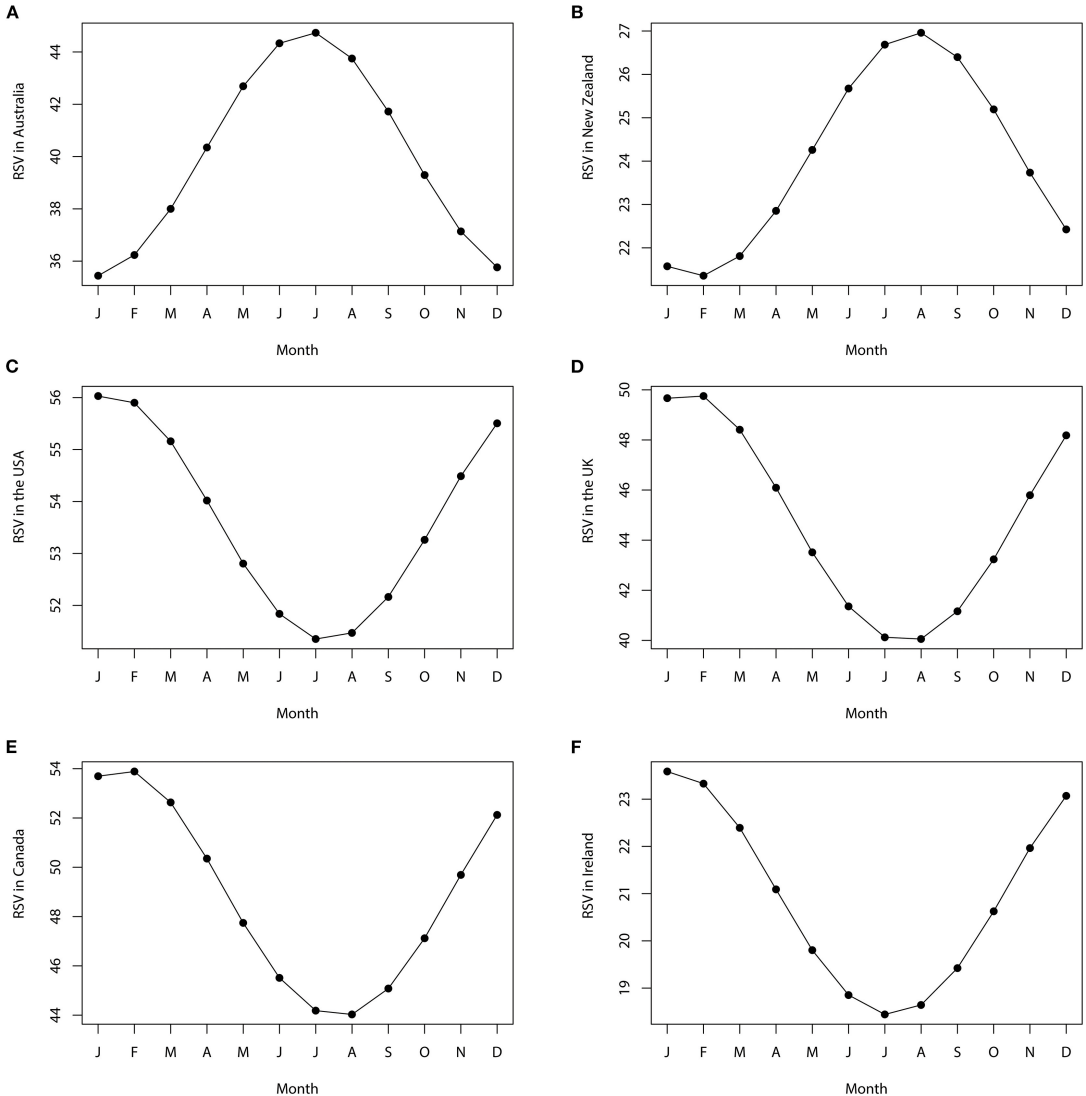
Plots of cosinor models for the seasonal variation of the Alzheimer's disease-related RSV in **(A)** Australia, **(B)** New Zealand, **(C)** the USA, **(D)** the UK, **(E)** Canada, and **(F)** Ireland from January 2004 to December 2020. The months are as follows: January, February, March, April, May, June, July, August, September, October, November, and December. RSV, relative search volume.

**Figure 5:**
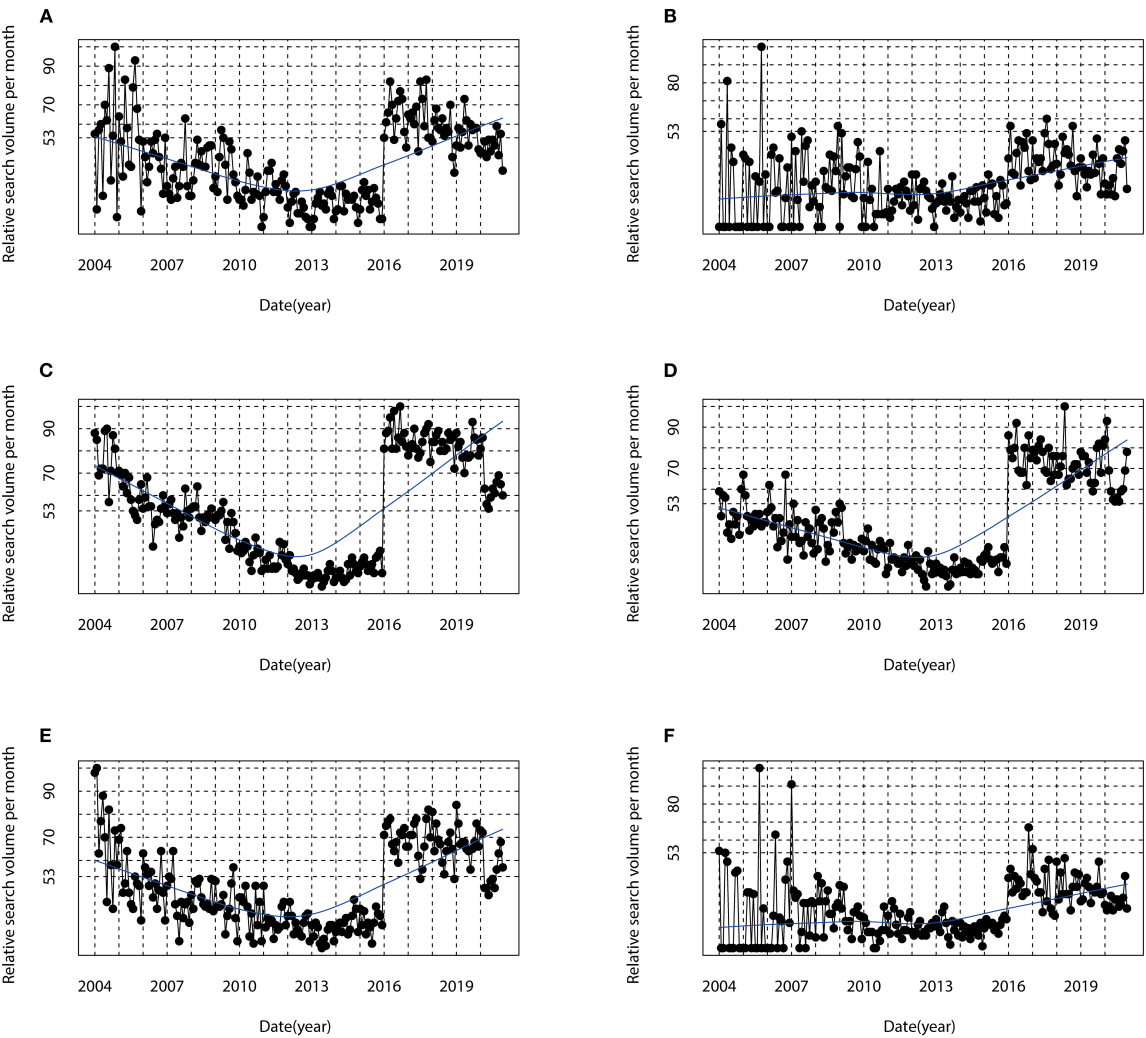
Time series plots for the relative search volume of [Alzheimer's disease] in **(A)** Australia, **(B)** New Zealand, **(C)** the USA, **(D)** the UK, **(E)** Canada, and **(F)** Ireland from January 2004 to December 2020.

The cosinor analyses showed a statistically significant seasonal variation in RSV for the term [Alzheimer's Disease] in Australia [amplitude (A) = 4.95, phase month (P) = 6.8, low point month (L) = 12.8, *P* < 0.05], New Zealand (A = 2.98, P = 7.8, L = 1.8, *P* < 0.05), the USA (A = 2.42, P = 1.3, L = 7.3, *P* < 0.05), the UK (A = 5.26, P = 1.6, L = 7.6, *P* < 0.05), Canada (A = 5.28, P = 1.6, L = 7.6, *P* < 0.05), and Ireland (A = 2.74, P = 1.2, L = 7.2, *P* < 0.05). The details are presented in Table 2. The seasonal variation curve fit with the “cosinor” model for the RSV is shown in Figure 4. Interestingly, regardless of the hemisphere, peaks were observed in the winter months (January for the northern hemisphere countries; June/July for the southern hemisphere countries) and trough in the summer months (July for the northern hemisphere countries; December/January for the southern hemisphere countries) (Figure 4, Table 2). Visual inspection of time series plots highlighted the consistency in the seasonal pattern that was observed in the cosinor analyses (Figure 5).

**Table 2:** The seasonal variation in the Alzheimer's disease-related relative search volumes.

**Country**	**Amplitude**	**Phase month^a^**	**Low point month^a^**	***p*-value**	**Time range**
Australia	4.95	6.8	12.8	<0.001	2004–2020
New Zealand	2.98	7.8	1.8	<0.001	2004–2020
USA	2.42	1.3	7.3	0.00127	2004–2020
UK	5.26	1.6	7.6	<0.001	2004–2020
Canada	5.28	1.6	7.6	<0.001	2004–2020
Ireland	2.74	1.2	7.2	<0.001	2004–2020

*Cosinor test was used to examine the seasonality*.

*Amplitude explains the size of the seasonal changes; phase month explains where the seasonal peak occurs; low point month explains where the seasonal low ebb occurs*.

a *Numeric values correspond to months as follows: January = 1, February = 2, March = 3, April = 4, May = 5, June = 6, July = 7, August = 8, September = 9, October = 10, November = 11, and December = 12*.

## Discussion

AD is one of the most famous diseases related to aging. With the progress of population aging, the medical burden of AD patients increases, and AD related research expands rapidly. The etiological theory of AD is gradually getting rid of the simple hypothesis of linear causality proposed by the original amyloid hypothesis, and more and more attention is paid to the important role of epigenetics, aging and lifestyle (16, 17), etc. Until now, all the drugs for treating AD can only improve the symptoms of AD, such as slowing the AD patient, etc., and cannot change the pathogenesis of the AD patient to cure AD. Therefore, finding ways to prevent and early diagnose AD and improving the health and living standards of patients through health care measures has become the focus of academic circles in recent years. There is growing evidence that GT can complement clinical and basic scientific data to inform patient and family consultation, public decision-making, and future clinical research (18–20). It is necessary to use GT analysis to understand the global public interest and seasonality of AD, which will contribute to clinical consultation services and etiology research of AD.

This study found that AD-related RSV declined steadily from January 2004 to December 2013 and rose steadily from January 2014 to December 2020. The increase in public interest in AD over the past few years may be related to the increase in the number of cases and deaths from AD (1, 3). Moreover, in the past decade, technological advances, such as the use of smartphones and the Internet, have enabled AD caretaker and their families to support AD information search and health management (21). Public access to health and wellness information through the Internet has increased dramatically. Several cohort studies based on European populations suggested that the prevalence of AD may have declined from the late twentieth century to the early twenty-first century (2, 22). This was probably due to the great economic, educational, and medical advances in Europe during that period. People's living environment and way of life have also improved considerably. This may explain the steady decline in AD-related RSV between January 2004 and December 2013. However, a study showed that between 1990 and 2010, the incidence of AD and dementia in China increased, and the burden of AD in low-and middle-income countries was severely underestimated (23). In terms of AD mortality, a study based on data from 2000 to 2010 in the United States shows that AD mortality has increased steadily over the past 30 years and is the sixth leading cause of death in the United States and the fifth leading cause of death in people 65 years and older, with mortality varying by age, sex, race, Hispanic origin, and geographic region (24). Regional and ethnic differences in AD prevalence have been recognized, which suggested that countries should pay more attention to the disease factors of specific ethnic and cultural groups when implementing AD related measures and researches (3, 23). Regional differences reportedly play a key role in dementia risk, with a higher risk in the north compared to the south in the northern hemisphere. The pattern in the southern hemisphere is more complex. Higher rates of dementia in the north compared to the south of Finland, England, Sweden, Scotland, Newfoundland, and China. This phenomenon is most likely the result of environmental risk factors such as sun exposure (and hence vitamin D levels) and air pollution (25). Higher levels of serum 25(OH)D was associated with a lower risk of dementia and AD (26). A randomized, double-blind, placebo-controlled trial found that daily oral vitamin D supplementation (800 IU/day) for 12 months may improve cognitive function and decrease Aβ-related biomarkers in elderly patients with AD (27). Our study also found that AD has a lower search heat in the southern hemisphere compared to the northern hemisphere. In Africa, in the northern hemisphere, AD search popularity is low, probably due to the fact that Africa's information and communications technology (ICT) infrastructure lags behind that of other regions (28). The effectiveness of ICT-enabled AD's prevention and intervention policies in Africa would then be limited by infrastructural conditions. This indicates that it is important to take management measures for AD based on geographical differences.

Online health information plays an important role in medicine and patients, and an increasing number of older people and their caregivers are turning to the Internet for health information (29). The potential for online information to have a significant impact on the relationship between patients and doctors, as well as their health, is also growing. The content and quality of AD prevention information currently available online is uneven (30). High-quality information can enable Internet users to make better decisions about their health, while low-quality information can not only lead to damage to patients' health but also worsen the relationship between older patients and doctors. More and more user-generated AD search information is expected to become a valuable resource for guiding health interventions. We can analyze and leverage this data to enable researchers to better understand user characteristics, information needs, and services for patients with AD and their caregivers. In GT, topics related to the characteristics and etiology of AD (Dementia, Memory, Cognitive, Agedness, Apolipoprotein E, Acetyl Choline), early onset AD (Early onset Alzheimer's disease), AD-related associations (Alzheimer's Disease and Related Disorders Association, ADRDA), care of AD patients (Caretaker), and diseases that can easily be confused with AD (Parkinson's disease, Vascular Dementia) have received special attention. Dementia describes a series of symptoms, such as memory loss, thinking difficulties, cognitive impairment, etc. Dementia includes many types, such as AD, vascular dementia, etc. ADRDA is an association that provides information and referral services for dementia patients (31). Parkinson's disease and AD belong to two common neurodegenerative diseases, some clinical symptoms and characteristics of the disease are similar, easy to be confused (32). Memory loss and cognitive difficulties are characteristic of AD, and the family and society burden of AD is heavy. Early-onset Alzheimer's disease is defined as AD that begins before age 65 and progresses rapidly, with the early onset of aphasia, dysgraphia, and apraxia (33). Good online health information and guidance from online doctors can effectively increase trust between doctors and patients (34). Physicians and health workers can conduct online and offline health information campaigns for AD patients and their caregivers based on global public interest.

This study provides evidence for seasonal variations in AD, with a peak in winter and a trough in summer. Cognitive impairment is an important disease characteristic of AD. It has been reported that the cognitive function of the human brain has an annual rhythm, or seasonality, which indicates that the seasons have a complex effect on brain function (35). Clinically, it has been found that there is a strong correlation between the seasons and cognition of older people. The average comprehensive cognitive function in summer and autumn was higher than that in winter and spring (36). This is accompanied by seasonal rhythms of AD-related proteins and specific genes in the cerebrospinal fluid. Seasonal changes in cognitive function of the brain are consistent with our findings. Interrelated diurnal and seasonal epigenetic and transcriptional rhythms may be an important feature of human brain biology. There are broad, site-specific and interrelated diurnal and seasonal rhythms of gene expression in the human brain (14). Seasonal factors may be important moderators of the association between cognition and AD pathology. These evidences provided the basis for the emergence of AD-related RSV seasonality in the results of this study. Although the important role of seasonal rhythms in human biological activities has been recognized, the evidence of AD seasonality is limited. This study provides new evidence that AD may be a seasonal disease. However, our judgment of AD seasonality is based on the condition that the change of Internet search quantity reflected the change of actual incidence, which may not 100% reflect the incidence of AD in real life. Therefore, seasonal variations in the incidence of AD based on hospital and community need to be further investigated. In summary, healthcare resources for AD can be strengthened during the season when they are most needed, both to identify the early stages of the disease earlier and to provide more support when patients are most vulnerable. Moreover, seasons should be considered an important confounding factor in the analysis of data from AD treatment trials and observational studies.

Several limitations of this study should be acknowledged. Firstly, we only included data from Google's search engine and did not include data from other search engines such as Yahoo and Baidu. Secondly, GT cannot provide the information of original search volume and users but only converted RSVs, so we could not obtain information on the user's age and sex for in-depth analysis. While analysis of specific user information can yield more valuable clues, Google's privacy policy protections limit that. Thirdly, quantifying public interest in AD and seasonal trends in interest changes through Internet queries does not actually represent seasonal changes in AD incidence. Seasonal variations in the incidence of AD based on hospital and community need to be further investigated. Despite these shortcomings, the data used in this study covered a wide geographical area and a long time span (17 years). This study quantifies public interest in AD and seasonal characteristics of interest changes by analyzing the frequency of Web queries via Google, and provides clues for follow-up institutional research on AD seasonality.

In summary, the global public interest in AD is increasing, and there is a significant seasonal trend, with a peak in winter and a low point in summer. Topics related to the characteristics and etiology of AD, early onset AD, AD-related associations, care of AD patients, and diseases that can easily be confused with AD are of the greatest concern to the global public. Understanding global public interest and the mechanisms underlying the seasonality of AD may help clinicians, policy makers and patients and their caregivers in the prevention, care, management and treatment of the disease.

## Data Availability Statement

The original contributions presented in the study are included in the article/supplementary material, further inquiries can be directed to the corresponding author/s.

## Author Contributions

Y-MM, X-YW, and D-QY conceptualized the study, participated in the study design. Y-MM and PW collected the data. Y-MM, PW, and X-YW conducted the statistical analysis. Y-MM wrote the manuscript. All authors contributed to manuscript revision, read, and approved the submitted version.

## Conflict of Interest

The authors declare that the research was conducted in the absence of any commercial or financial relationships that could be construed as a potential conflict of interest.

## Publisher's Note

All claims expressed in this article are solely those of the authors and do not necessarily represent those of their affiliated organizations, or those of the publisher, the editors and the reviewers. Any product that may be evaluated in this article, or claim that may be made by its manufacturer, is not guaranteed or endorsed by the publisher.
